# Exploring Different Strategies for Efficient Delivery of Colorectal Cancer Therapy

**DOI:** 10.3390/ijms161125995

**Published:** 2015-11-11

**Authors:** Congcong Lin, Huei Leng Helena Ng, Weisan Pan, Hubiao Chen, Ge Zhang, Zhaoxiang Bian, Aiping Lu, Zhijun Yang

**Affiliations:** 1 School of Chinese Medicine, Hong Kong Baptist University, 7 Baptist University Road, Kowloon Tong, Hong Kong, China; 14485680@life.hkbu.edu.hk (C.L.); hueilengng@yahoo.com (H.L.H.N.); hbchen@hkbu.edu.hk (H.C.); zhangge@hkbu.edu.hk (G.Z.); bzxiang@hkbu.edu.hk (Z.B.); 2 Department of Pharmaceutics, School of Pharmacy, Shenyang Pharmaceutical University, 103 Wenhua Road, Shenyang 110016, China; ppwwss@163.com; 3 Changshu Research Institute, Hong Kong Baptist University, Changshu Economic and Technological Development (CETD) Zone, Changshu 215500, China

**Keywords:** colorectal cancer, chemotherapy, drug delivery system, colon-specific drug delivery system, systemic drug delivery system

## Abstract

Colorectal cancer (CRC) is the third most common cancer and the fourth leading cause of cancer death in the world. Currently available chemotherapy of CRC usually delivers the drug to both normal as well as cancerous tissues, thus leading to numerous undesirable effects. Much emphasis is being laid on the development of effective drug delivery systems for achieving selective delivery of the active moiety at the anticipated site of action with minimized unwanted side effects. Researchers have employed various techniques (dependent on pH, time, pressure and/or bacteria) for targeting drugs directly to the colonic region. On the other hand, systemic drug delivery strategies to specific molecular targets (such as FGFR, EGFR, CD44, EpCAM, CA IX, PPARγ and COX-2) overexpressed by cancerous cells have also been shown to be effective. This review aims to put forth an overview of drug delivery technologies that have been, and may be developed, for the treatment of CRC.

## 1. Introduction

Colorectal cancer (CRC) is the third most commonly diagnosed cancer worldwide, accounting for 10% of all cancers and 1.36 million new cases with over half a million deaths every year [1]. Around 55% of the cases were diagnosed in developed regions with the highest rates in Australia/New Zealand [2]. CRC mainly affects the elderly with a median age of onset of about 69 years old [3]. Many survivors must cope with long-term sequelae of treatment as well as psychological concerns such as fear of recurrence. 

The current mainstay of treatment for CRC is surgical resection followed by adjuvant chemotherapy. Unfortunately, to date, the chemotherapy is far from optimal, resulting in objective responses in only 30% of cases [4]. Traditional treatment regimens for CRC are involved in delivering the drug to both tumour and normal tissue, resulting in unexpected side effects such as neutropenia, anemia, hand-foot syndrome, diarrhea, gastrointestinal toxicity, mucositis, nausea, vomiting, fatigue, hematologic disorders and liver toxicity [5,6]. Although in 2004, Bevacizumab (a humanized recombinant monoclonal antibody against vascular endothelial growth factor-A) was successfully introduced as a therapeutic regimen in combination with first- and second-line treatment of metastatic colorectal cancer (mCRC) and led to improvement in progression-free survival [7,8], unfortunately it failed to exhibit a positive impact for the outcome of overall survival. 

Therefore, alternative therapeutic strategies for efficient drug delivery to tumour sites, but not normal organs or tissues are a desperate need. Firstly, the limitations of current drugs in the treatment of CRC are addressed in this review. Then, we assess the various formulation approaches that have been investigated for colon-specific delivery of drugs used in the treatment of CRC and highlight some specific targets for targeting therapy by systemic drug delivery. The objective of this review is to provide some potential directions that may drive the development of chemotherapy for patients with CRC.

## 2. Colorectal Cancer and Its Current Treatment Regimen

Colorectal cancer often develops over a period of 10–15 years [9]. It largely originates from adenomas, a group of benign, noncancerous colonic polyps. As a consequence of mutations in tumour suppressors, in apoptotic genes and oncogenes, about 10% of the adenomas will progress and develop into cancers [10]. Despite that a greater understanding of the molecular basis of CRC has been achieved with the development of current gene-identification techniques, as shown in Table 1, these research results have failed to make substantial improvement in outcomes in CRC patients. Thus, more promising strategies of drug delivery systems to achieve better outcomes are needed.

**Figure 1:**
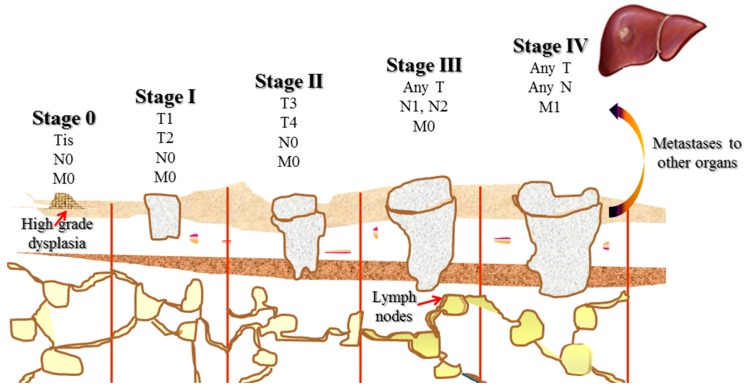
Classification of colorectal cancers (American Joint Commission on Cancer): Tis—carcinoma *in situ*: intraepithelial or invasion of lamina propria; T1—tumour invades submucosa; T2—tumour invades muscularis propria; T3—tumour invades through muscularis propria into subserosa or into nonperitonealized pericolic or perirectal tissues; T4—tumour penetrates the surface of the visceral peritoneum or tumour directly invades or is histologically adherent to other organs or structures; N0—no regional lymph node metastasis; N1—metastasis in one to three regional lymph nodes; N2—metastasis in four or more regional lymph nodes; M0—no distant metastasis; M1—distant metastasis.

Classification of cancer by anatomic disease extent is one of the most important factors for prognosis and therapeutic decision. Based on TNM (T: primary tumour site; N: regional lymph node involvement; M: presence or otherwise of distant metastatic) and UICC (Union for International Cancer Control) staging system (Figure 1), CRC can be treated by surgery, chemotherapy, radiation, immunotherapy or palliative care [11]. Surgical resection offers high cure rates for CRC in early stages, of which the success rates are 90% and 75% for Stage I and II CRC respectively [12]. Fortunately, no additional treatment is required for patients with stage I CRC following surgical procedure as they have little benefit from additional treatment with low recurrence rate (about 3%). However, adjuvant chemotherapy is recommended for all individuals with stage III CRC, which have a higher risk of relapse at about 60% [13]. Chemotherapy can be added as post-surgery adjuvant, pre-surgery neo-adjuvant or as primary therapy to inhibit tumour cell growth, induce cell apoptosis or decrease metastasis opportunity [14]. Although there has been a remarkable advance in chemotherapy for CRC patients in the past two decades, stage IV disease is usually incurable [15]. The best remedy for the patients with metastatic CRC (mCRC) is to improve the quality of life by improving systemic treatment [16].

At present, several drugs are available for the management of CRC. 5-Fluorouracil (5-FU)/Leucovorin is the first-line treatment, and the most common chemotherapy for metastatic CRC by inhibiting thymidylate synthase [17,18]. With the evolution of chemotherapy, Saltz *et al.* [19] found that treatment with a combination of Irinotecan (Camptosar, Pharmacia), a potent inhibitor of topoisomerase I, 5-FU, and Leucovorin resulted in significantly longer progression-free survival (median, 7.0 *vs.* 4.3 months; *p* = 0.004), greater confirmed response (39% *vs.* 21%, *p* < 0.001), and longer overall survival (median, 14.8 *vs.* 12.6 months; *p* = 0.04) than 5-FU/Leucovorin alone. An extensive body of data shows that, Fluoropyrimidines, Irinotecan and Oxaliplatin have emerged as cornerstones of chemotherapy for CRC. However, these traditional pharmaceutical therapeutic regimens are usually accompanied by severe mucositis, myelosuppression, and cumulative neurosensory toxicity and hematological adverse reactions due to unspecific distribution into intestinal mucosa, bone marrow, liver and other healthy tissues [20,21,22]. The cumulative toxicity such as neurosensory toxicity by Oxaliplatin may require the patient to withdraw from treatment [23]. Although Bevacizumab, a monoclonal antibody targeting angiogenesis, and Cetuximab or Panitumumab, both monoclonal antibodies targeting EGFR, have lately been added to the arsenal of treatment candidates for colorectal carcinoma [24,25,26], they provide a relatively small improvement on survival outcomes. Therefore, design of alternative drug delivery systems to minimize toxicity and improve the tumour targeting specificity of CRC is gaining significant interest in the scientific community.

**Table 1:** Genomic Instability in Colorectal Cancer.

Type of Genes	Type of Instability	Genes Involved	Frequency (%)	Comments
Tumour suppressor genes	Chromosomal instability	*APC*	85	Somatic mutations inactivating both copies of *APC* are present in most sporadic colorectal cancers; A germ-line mutation in familial adenomatous polyposis with an 80% to 100% lifetime risk of colorectal cancer. Activation of Wnt signaling due to inability to degrade the β-catenin oncoprotein [27,28,29,30,31].
		*PTEN*	10–15	Germ-line mutations that promote activation of PI3K pathway signaling through loss of function [32,33,34].
		*TP53*	35–55	Germ-line mutation in Li-Fraumeni syndrome; inactivates missense mutations pairs with loss of heterozygosity at 17p [35,36,37].
		*SMAD4*	10–35	Germ-line mutation in approximately 40% of juvenile polyposis; a critical component of transforming growth factor β signaling pathway; inactivated by homozygous or mutation with loss of heterozygosity at 18q [38,39,40,41].
	DNA mismatch-repair defects	*MLH1*, *MSH2*, *MSH6 MYH*	15–25	Germ-line mutation permitting the accumulation of oncogenic mutations and tumour suppressor loss [42,43,44].
	Aberrant DNA methylation	*MLH1*	15	Silencing of the promoter region of the genes in mismatch-repair system by hyper-methylation of CpG islands [45,46].
Oncogenes	DNA mismatch-repair defects	*RAS*, *BRAF*	13–37	Activates the mitogen-activated protein kinase signaling pathway [47,48,49].

## 3. Exploring Drug Delivery to Colorectal Cancer

Accordingly, exploring a better drug delivery system for chemotherapy is a must to increase the life expectancy of the CRC patient. Based on the specific property of CRC, targeted delivery of the active moiety at the anticipated site may be achieved by colon-specific as well as systemic drug delivery.

### 3.1. Colon-Specific Drug Delivery System of Colorectal Cancer

A colon-specific delivery system has attracted considerable attention for its potential effectiveness in carrying agents such as 5-FU, Oxaliplatin, Capecitabine and Irinotecan for both localized and systemic therapy. Furthermore, the success of delivering peptide and protein, such as Bevacizumab, Cetuximab, Panitumumab [50,51], by colonic delivery also makes it a potential strategy for achieving molecularly targeted therapies of CRC. Colonic delivery can be accomplished by oral or direct administration from the rectum. However, only a small amount of the drug administered rectally would reach the splenic flexure and the treatment is not convenient for the patient. Therefore, most of the colon-specific drug delivery systems utilize the oral route and this will be the main focus of this review. In order to achieve successful colonic delivery, the unique physiological condition of the colon must be considered. Furthermore, the upper gastro-intestinal (GI) physiology and the transit of pharmaceuticals through these regions also play an important part in achieving site specificity.

Anatomically, the GI tract is divided into stomach, small intestine and large intestine. The colon is about 1.5 m long with a surface area of 0.3 m^2^ resulting in a lower absorption capacity than that of the small intestine (6 m in length and surface area ~120 m^2^) [52]. Consequently, the drug has to surmount the barriers posed by the luminal environment before coming into contact with the colonic epithelium. Based on the various physiological properties of the GI tract (Table 2), efforts can be made on the following four aspects.

**Table 2:** Physiological Properties of the Gastrointestinal Tract *

Organ	pH	Transit Time (h)	Bacterial Count (CFU/mL)
Stomach	2–3	<1 (fasting), >3 (fed)	10^2^–10^4^
Small intestine	6.5–7	3–4	10^3^–10^4^
Large intestine	7–8	>20	10^11^–10^12^

* Data taken from [53,54,55,56].

#### 3.1.1. pH-Dependent Systems

The pH increases progressively from the stomach (pH 2–3), small intestine (pH 6.5–7) to the colon (pH 7–8) [53]. Application of pH-dependent polymers is based on these differences in pH levels of the tract. The most commonly utilized polymers are methyl methacrylate and methacrylic acid copolymers (Eudragit^®^) that dissolve with the pH range of 5.5–7.0 [57]. A 5-FU entrapped methacrylic-base copolymer nanogel was prepared and presented to be an effective approach targeting to colon *in vitro* [58]. In fact, a number of preparations are commercially available, for instance, mesalazine (Pentasa^®^, Asacol^®^, Salofalk^®^) and budesonide (Entocort^®^) for treatment of ulcerative colitis and Crohn’s disease. However, studies by Rijk *et al.* demonstrated that several brands of the pH-dependent mesalazine tablets showed significant individual variations in urinary recovery of the drug [59]. This is due to the fact that the pH of GI varies between and within individuals [60]. Furthermore, a pH-dependent polymer coated system also showed a tendency to release their drug prior at the duodenum [61]. Other factors such as composition of GI fluid, status of feeding and time of transition at the ileocaecal junction will also lead to poor site specificity or even the failure of pH-dependent systems [62,63].

#### 3.1.2. Time-Dependent Systems

The rough estimated transit times in healthy humans following ingestion of a standard meal (*i.e.*, solid, mixed foods) of stomach, small intestine, and large intestine are 3–5 h, 3–4 h and 30–40 h, respectively [54]. Time-dependent systems are based on the drug’s duration of travel between ingestion and colonic arrival. A Chronotopic™ system of this principle was established with regard to colon-specific targeting. The system is composed of a drug-containing core coated with a swellable hydrophilic polymer such as hydroxypropylmethylcellulose, which is capable of delaying the release of drugs through slow interaction with the biological fluids [64]. One challenge to this system is that the gastric emptying time is highly variable and this may lead to early drug release in the small intestine or a deferred release of drug far down in the transverse colon [65]. In order to overcome the huge variability in gastric emptying time, a gastroresistant film is applied onto the Chronotopic™ system, which is expected to protect the integrity of the dosage as long as it is located in the stomach [64]. This design is based on the assumption that gastroresistant film dissolves rapidly in the small intestine. However, this assumption is in conflict with gamma scintigraphy studies: dissolving and disintegration of the gastroresistant film will take up to 2 h in the human small intestine [66,67,68]. In addition, large single-unit dosage forms are held in the ileocaecal junction for extended periods, which also vary in the range of 2–10 h [69]. Taking account of these effects, the prediction of arrival time of a dosage form will be challenging.

#### 3.1.3. Pressure-Dependent Systems

As a result of a spasmodic intense peristaltic motility, higher intraluminal pressure is built up in the colon than in the small intestine. The colon luminal pressure is approximately 2.0 newtons [50]. Pressure-dependent systems such as pressure-controlled colon delivery capsules are designed according to this principle. They are prepared by coating the inner surface of gelatin capsule with a water-insoluble polymer such as ethyl cellulose (EC) and filling the capsule with suppository base such as polyethylene glycol (PEG). The PEG base dissolves at body temperature followed by the immediate dissolution of the gelatin layer, resulting in the formation of an “EC balloon”. When exposed to the increased viscosity and pressure of the colonic lumen, the balloon then disintegrates and liberates the loaded drug [70,71]. However, the variability of fluid volume that comes in contact with the capsule, as well as the varying motility of the colon proves problematic in this kind of colonic delivery system [72].

#### 3.1.4. Bacteria-Dependent Systems

The bacterial count has been estimated to be over 10^11^ per gram in the colon which accounts for almost one-third of the dry weight of feces in man, compared with 10^4^ per gram in the duodenum [55,56]. These vast colonic microfloras consist of mainly anaerobic bacteria that secrete biodegradable enzymes including glucoronidase, xylosidase, arabinosidase, galactosidase, nitroreductase, and azoreductase, which is only present in the colon. Therefore, this unique feature of the colon can be exploited to target colonic drug release. Natural polysaccharides such as chitosan, pectin, chondroitin sulphate, cyclodextrin, dextrans, guar gum, inulin, amylose, and locust bean gum are ideal candidates based on this principle. These nontoxic molecules can avoid degradation in the upper tract, but are used as a substrate by the anaerobic bacteria in the colon resulting in the site-specific drug release [73,74,75]. A new orally-administered 5-FU tablet was prepared by compression coating technique using granulated chitosan. A study in beagle dogs showed a formula with 50 mg coat weight per tablet exhibited the best protection profile, where <10% of the drug released after 6 h demonstrated the potential for colon targeting [76]. However, these polysaccharides are associated with several limitations. They are lacking in good film forming properties or swelling tendency in aqueous media. Another promising attempt is the import of prodrugs to this bacterially triggered system. The prodrug is a pharmacologically inactive derivative of a parent drug molecule that is designed to release the active moiety by enzymatic hydrolysis in the colon. One of the most susceptible linkages to bacterial hydrolysis in the colon is the azo linkage. In the case of sulfasalazine, the active moiety of 5-aminosalicylic acid (5-ASA) was coupled with sulfapyridine by azo bonding. This system has successfully delivered 5-ASA in intact form to the colon [77]. Since a functional group is essential for this concept, an alternative solution is the application of azopolymer [78,79]. However, the poor film-forming properties and the risk of formation of toxic by-products of azopolymers limited its development [80].

**Table 3:** Different Colon-specific Drug Delivery System Approaches for Colorectal Cancer.

Approach	Designed Principle	Examples	Comments
pH-dependent systems	The pH increases progressively from the stomach (pH 2–3), small intestine (pH 6.5–7) to the colon (pH 7–8) [53].	A 5-FU entrapped methacrylic-base copolymer nanogel was prepared and presented to be an effective approach targeting to colon *in vitro* [58].	The pH of GI varies between and within individuals [60]. Other factors such as composition of GI fluid, status of feeding and time of transition at the ileocaecal junction will also lead to the poor site specificity or even the failure of pH-dependent systems [62,63].
Time-dependent systems	The rough estimated transit times in healthy humans following ingestion of a standard meal (*i.e.*, solid, mixed foods) of stomach, small intestine, and large intestine are 3–5 h, 3–4 h and 30–40 h, respectively [54].	A Chronotopic™ system composed of a drug-containing core coated with hydrophilic and gastroresistant polymer was built up and provided a successful break-up in the colon [64].	The gastroresistant film cannot dissolve rapidly in the small intestine, and will take up to 2 h [66,67,68]. In addition, large single-unit dosage forms are found to be held in the ileocaecal junction for extended periods which will be the challenge of the prediction of dosage arriving time [69].
Pressure-dependent systems	The intraluminal pressure in the colon is higher than in the small intestine due to the spasmodic intense peristaltic motility [50].	A novel EC-coated gelatin capsule was prepared and evaluated in healthy male human volunteers which indicated its ability of colon delivery of drug [70].	The data on luminal pressures in different region of the GI is limited. In addition, the variability of fluid volume and varying motility of colon will be challenging in pressure-dependent systems [72].
Bacteria-dependent systems	The bacterial count is over 10^11^ per gram in the colon compared with 10^4^ per gram in the duodenum [55,56]. These vast colonic microfloras secrete a diverse array of enzymes which are only present in colon.	A new orally-administered 5-FU tablet was prepared by compression coating technique using granulated chitosan and demonstrated its potential for colon targeting by a study in beagle dogs [76]; A prodrug of 5-ASA was developed and successfully delivered 5-ASA in intact form to colon by azo bonding [77].	The poor film-forming properties of nontoxic molecules, specific requirement of functional group and the risk of toxic by-products formation of azopolymer limitied the development of bacteria-dependent systems [80].

As summarized in Table 3, every type of colonic systems has its weakness. Herein, attempts have been made to develop multiparticulate drug delivery systems by combining two or more mechanisms. Colon-targeted celecoxib-loaded microparticles using a time-dependent and pH-dependent coating system was successfully prepared for CRC by Ghorab *et al.* [81]. It was demonstrated that the double coating provides a satisfactory protection for colonic targeting, and the microparticles elevated the bioavailability of the drug and extended the duration of drug-plasma concentration in rats. In another study, a novel 5-FU oral formulation using pH-enzyme Di-dependent chitosan microspheres has been investigated for its colon targeting efficiency [82]. The drug release behaviors *in vitro* have shown that the drug was protected from being released in the upper gut, and pharmacokinetics study *in vivo* revealed its potential for colon-specific drug delivery of 5-FU. Therefore, exploration of multiparticlulate drug delivery systems may be promising approaches for CRC.

### 3.2. Systemic Drug Delivery System of Colorectal Cancer

**Figure 2:**
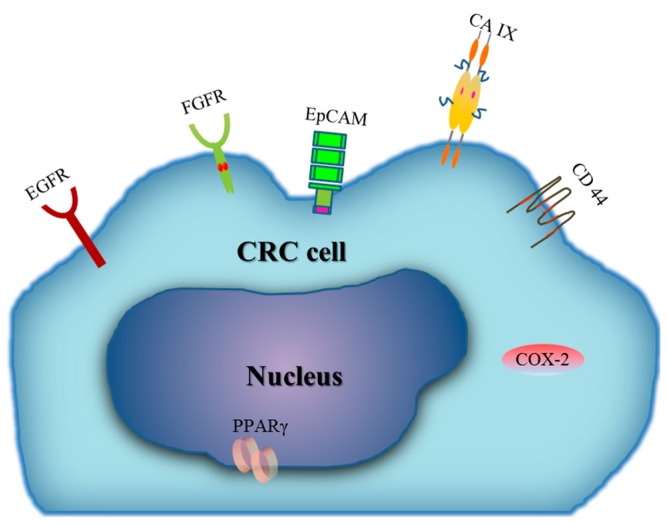
Proteins overexpressed in CRC cell. EGFR: Epidermal growth factor receptor; FGFR: fibroblast growth factor receptor; CD44: Cluster of differentiation 44; EpCAM: Epithelial cell-adhesion molecule; CA IX: Carbonic anhydrase IX; PPARγ: Peroxisome proliferator-activated receptor γ; COX-2: Cyclooxygenase-2.

Approximately 35% of patients with CRC have metastases upon diagnosis [83]. Liver and lung are the most frequent sites of dissemination of CRC [84,85]. Unfortunately, most of these metastases are unresectable due to involvement of non-resectable structures [83]. Thus, there is a great need to develop systemic chemotherapy treatments aiming to lessen symptoms for CRC due to this grim reality. However, how to maximally accumulate drug to the tumour site is still the main challenge to researchers. Since “passive targeting” based on the enhanced permeation and retention (EPR) effect of the tumour can only accumulate a very small fraction (<5%) of the total administered formulation to the intended target site [86], researches have focused on “active targeting” of the drug to the tumour. Therefore, searching for specific proteins that are over-expressed by CRC cells and directly inhibiting their function may be the other efficient way to minimize the toxicity to normal tissues. Below we summarize some proteins that are overexpressed in CRC (Figure 2) which could be the candidate targets for drug treatment.

#### 3.2.1. Epidermal Growth Factor Receptor (EGFR)

Epidermal growth factor receptor (EGFR) is a 170-kDa transmembrane glycoprotein that consists of an intracellular tyrosine-kinase domain, a transmembrane lipophilic segment and an extracellular ligand binding domain [87]. EGFR mediates signaling by the transfer of phosphate molecules from ATP to an active site of tyrosine kinase, triggering cascade of MAPK and PI3K that will protect cells from apoptosis, facilitate invasion and promote angiogenesis reaction [88]. EGFR has been found to be overexpressed in CRC patients (expression rates 25% to 82%) [89]. Interestingly, the EGFR reactivity rate was 97% in the study reported by Spano *et al.* [90]. In this context, the EGFR-directed monoclonal antibodies (Cetuximab and Panitumumab) were successfully developed against CRC. These antibodies showed a significant improvement in progression-free survival [91,92]. However, it has been shown that patients with *KRAS* and *NRAS* mutations resulted in no or lower response rates to Cetuximab and Panitumumab [93,94]. Therefore, the examination of these genes in CRC patients is recommended in order to identify patients appropriate to be treated with anti-EGFR antibodies. EGFR-targeted drug delivery system has also been studied, for example, anti-EGFR immunoliposomes modulated with Fab’ fragments of Cetuximab were constructed by Mamot *et al.* [95]. The immunoliposomes were internalized extensively within tumour cells (92% of analyzed cells *vs.* <5% for nontargeted liposomes), and treatment with anti-EGFR immunoliposome-doxorubicin produced substantial tumour regressions and was the most efficacious treatment in both the EGFR-overexpressing tumour model featuring MDAMB-68 human breast tumour and U87 human glioblastoma xenografts as compared to non-targeted liposomes or free doxorubicin. This finding implies that loading chemotherapy drug in the EGFR targeted drug delivery system may be an alternative option for the treatment of CRC.

#### 3.2.2. Fibroblast Growth Factor Receptor (FGFR)

The fibroblast growth factor receptor (FGFR) family consists of four members, named FGFR1, 2, 3, and 4 which contains an extracellular ligand domain composed of three immunoglobulin-like domains, a single transmembrane helix domain, and an intracellular domain with tyrosine kinase activity. These receptors bind fibroblast growth factors (FGFs), members of the largest family of growth factor ligands [96]. FGF-FGFR binding activates the intracellular Ras/MAPK signaling cascade which is essential for growth factor-induced cell proliferation and differentiation signaling cascades [97]. Patients with CRC have been reported to overexpress all four FGFRs [98]. In a CRC tumour xenograft mouse model, combined therapy with a recombinant FGFR1 protein vaccine and low-dose gemcitabine suppressed tumour growth and antiangiogenesis was present [99]. The invasive front of the cancer cells exhibited a stronger C2 (one of the variants of FGFR2) expression than the surface areas of the cancers, while FGFR2 was not detected in the non-tumourous mucosa of peripheral CRC lesions. Besides that, FGFR2 was detected in the cytoplasm of adenocarcinomas. Furthermore, it was reported that shRNA-targeting FGFR2 in CRC cell lines inhibited cancer cell growth, migration, and invasion [100]. Chen *et al.* designed a truncated form of human basic fibroblast growth factor peptide (tbFGF) which functioned as a FGFR ligand, and successfully attached it to the surface of cationic liposomal doxorubicin (LPs-DOX) and paclitaxel (LPs-PTX) [101]. In this study, the FGFR-mediated LPs-PTX achieved 7.1-fold accumulation of paclitaxel in tumour tissue than those of free paclitaxel in melanoma cells of B16 tumour-bearing mice. Furthermore, tbFGF-LPs-DOX and tbFGF-LPs-PTX both showed significant inhibition of tumour growth, and improvement in survival rate of tumour-bearing mice as compared with mice treated with free and liposomal drugs. These results suggest that FGFR might be a novel therapeutic target for CRC.

#### 3.2.3. Cluster of Differentiation 44 (CD44)

Cluster of differentiation 44 (CD44) is a transmembrane cell adhesion molecule, which generally acts as a specific receptor for hyaluronic acid [102]. It consists of an N-terminal extracellular binding region for hyaluronic acid, a membrane proximal region for insertion of the variant exons (v1–10), a transmembrane domain, and cytoplasmic binding sites for ankyrin and ERM (ezrin, radixin, moesin) proteins [103]. Several CD44 variants have been found to be overexpressed in CRC specimens [104]. CD44 plays a major role in the regulation of cell adhesion, growth, differentiation, migration and angiogenesis, and contributes to tumour progression by promoting invasion and metastasis. Knockdown of CD44 by lentiviral RNA interference in primary colon cancer cell lines reduced clonogenicity *in vitro* and tumourigenicity *in vivo* [105]. A doxorubicin hydrochloride (Dox) loaded CD44-targeted drug delivery system based on hyaluronic acid modified mesoporous silica nanoparticles (MSNs) has been developed and shown greater cytotoxicity to HCT-116 (human colon cancer cells) than free Dox and nontargeted MSNs [106]. In these respects, CD44 could be a potential therapeutic target for the treatment of CRC.

#### 3.2.4. Epithelial Cell-Adhesion Molecule (EpCAM)

Epithelial cell-adhesion molecule (EpCAM), a 40 kDa glycoprotein [107], can undergo regulated intra-membrane proteolysis leading to release of its small intracellular domain EpICD. Subsequently, the released EpICD will combine with adaptor proteins FHL2 and β-catenin ultimately leading to formation of a large nuclear complex containing transcription factor LEF/TCF, which can turn on transcription of c-myc and cyclin genes and thereby drive cancer and stem cell proliferation [108]. As one of the earliest tumour markers, EpCAM is expressed in 85% of CRC [109]. A pivotal trial in patients with surgically resected Dukes’ stage C CRC, in which the patients were randomized to observation or treatment with Edrecolomab (anti-EpCAM antibody), Edrecolomab treatment showed a significant reduction of recurrence and death rate, and a benign safety profile [110,111]. In the study by Li *et al.*, curcumin-loaded lipid-polymer-lecithin hybrid nanoparticles (CUR-NPs) were synthesized and functionalized with RNA Aptamers (Apts) against EpCAM for targeted delivery to CRC cells [112]. A substantial improvement in cell binding, cellular uptake and cytotoxicity was achieved toward HT29 colon cancer cells with Apt-CUR-NPs. These results indicate that the EpCAM targeted delivery of novel chemotherapeutic agents could be a promising therapeutic strategy for CRC.

#### 3.2.5. Carbonic Anhydrase IX (CA IX)

Carbonic anhydrase IX (CA IX) is from a family of zinc metalloenzymes, and functions as an important component of pH-regulating machinery that is activated in response to hypoxia [113]. Hypoxia is a salient feature of many types of solid tumours [114]. In metabolism, CA IX catalyzes the reversible hydration of pericellular carbon dioxide (CO_2_) to bicarbonate (HCO_3_^−^ and protons (H^+^) (CO_2_ + H_2_O ↔ HCO_3_^−^ + H^+^), which has been proposed to contribute to cellular alkalinization, and promote cell survival and growth through intracellular pH maintenance. The increasingly acidic extracellular microenvironment results in the death of non-tumour cells and accelerates degradation of the extracellular matrix, thereby promoting the invasion and proliferation of acid-resistant cancer cells [115]. Colorectal tumours have shown an abnormal CA IX expression, especially in high proliferation areas [116]. Furthermore, CA IX was found to be the most upregulated gene in colorectal cancer samples studied by cDNA microarray [117]. Previous studies have demonstrated that CA IX-directed immunoliposomal docetaxel exhibited the strongest growth inhibitory effect against CA IX-positive lung cancer cells when compared with non-targeted liposomal docetaxel or free docetaxel solution [118]. In these regards, we propose that CA IX can be an attractive option for the therapeutic targeting of CRC.

#### 3.2.6. Peroxisome Proliferator-Activated Receptor γ (PPARγ)

Peroxisome proliferator-activated receptor γ (PPARγ) is a member of the nuclear receptor superfamily including receptors for steroids, thyroid hormone, vitamin D and retinoic acid [119]. Although PPARγ is well known for its function in adipocyte gene expression, insulin sensitivity and lipogenesis [120], other roles for modulating the growth and differentiation of colon cancer cells have also been discovered. Sarraf *et al.* [121] analyzed the expression of human PPARγ mRNA in normal colonic epithelium and colon tumours from the same patients, and showed that all of the colorectal tumours analyzed (11 of 11) had a high level of PPARγ mRNA as well as in the normal colon tissue adjacent to the tumour. In addition, a significant reduction in tumour volume between colon tumours xenograft mice given troglitazone (a specific ligand for PPARγ) *vs.* those given vehicle was observed. Although there are few studies on the targeted drug delivery approaches of PPARγ, the relatively non-toxic nature of PPARγ ligands like troglitazone and many others in development indicate that they should be a new approach for CRC therapy [121].

#### 3.2.7. Cyclooxygenase-2 (COX-2)

Cyclooxygenase-2 (COX-2) is a member of cyclooxygenase family that catalyzes the conversion of arachidonic acid to prostaglandins [122]. Prostaglandin E2 (PGE2) being the predominant product of COX-2 inhibits apoptosis and stimulates tumour growth and angiogenesis via stimulation of β-catenin/T-cell factor dependent transcription [123]. It has been reported that over 50% of colorectal adenomas and 80%–90% CRC overexpress COX-2 [124]. This enzyme is generally not found in healthy colon tissue, but is thought to fuel abnormal cell growth. Studies of celecoxib (a COX-2 inhibitor) have demonstrated that it can effectively decrease the number and size of colon polyps with as short as six months of treatment [125], and also appear to be beneficial for breast cancer [126]. In addition, inhibition of COX-2 by celecoxib delayed tumour growth and metastasis in xenograft human colon tumour models [127]. These published experimental studies imply the possible approach of developing a drug delivery system targeting COX-2 in CRC patients.

## 4. Conclusions

In this review, we have summarized some novel drug delivery systems by virtue of the specific physiological condition of the luminal environment, and presented some potential specific molecular targets for CRC treatment. In terms of colonic delivery, the unique pH, transit time, pressure and bacteria properties of the GI tract provide both opportunities and barriers. Therefore, a multi-particulate drug delivery system by combination of two or more mechanisms would be a promising way for CRC treatment. For systemic drug delivery, targeting specific overexpressed proteins have been the main fundamental basis for the design of drug delivery system. The overexpressed proteins such as FGFR, EGFR, CD44, EpCAM, CA IX, PPARγ and COX-2 can be attractive targets.

In cancer therapies, the ideal drug delivery system is the one that places the drug only at the target tumour cell. Although there are many exciting new avenues in drug targeting both in colonic and systemic treatment for CRC, the ideal scenario is still beyond our grasp. In reality, to effectively target drugs to the tumour cell, the prepared drug delivery system has to fulfill four vital requirements: retain, evade, target and release [128]. The heterogeneity of cancer cells also complicates the issue. Thus, all of these factors, and many others that remain unknown, should be taken into consideration for developing better drug delivery for CRC. Unfortunately, current chemotherapeutic treatment for CRC uses high doses of cytotoxic medicaments, specifically adjuvant combinations of 5-FU and Irinotecan, which result in adverse effects to the affected patient. It is necessary to develop new nanomedicines with multifunctional characters that bring together different chemotherapeutic agents; ideally this would allow double or triple therapies with lower systemic doses, and significantly reduce undesirable side-effects.
